# Comparative Genomics of Plasmid-Bearing *Staphylococcus aureus* Strains Isolated From Various Retail Meats

**DOI:** 10.3389/fmicb.2020.574923

**Published:** 2020-10-23

**Authors:** Anand B. Karki, Leena Neyaz, Mohamed K. Fakhr

**Affiliations:** Department of Biological Science, The University of Tulsa, Tulsa, OK, United States

**Keywords:** *S. aureus*, plasmids, retail meat, comparative genomics, whole genome sequencing

## Abstract

Food poisoning due to the consumption of *Staphylococcus aureus* contaminated food is a major health problem worldwide. In this study, we sequenced the genomes of ten plasmid-bearing *S. aureus* strains isolated from retail beef, chicken, turkey, and pork. The chromosomes of the strains varied in size from 2,654,842 to 2,807,514 bp, and a total of 25 plasmids were identified ranging from 1.4 to 118 kb. Comparative genomic analysis revealed similarities between strains isolated from the same retail meat source, indicating an origin-specific genomic composition. Genes known to modulate attachment, invasion, and toxin production were identified in the 10 genomes. Strains from retail chicken resembled human clinical isolates with respect to virulence factors and genomic islands, and retail turkey and pork isolates shared similarity with *S. aureus* from livestock. Most chromosomes contained antimicrobial resistance, heavy metal resistance, and stress response genes, and several plasmids contained genes involved in antimicrobial resistance and virulence. In conclusion, the genomes of *S. aureus* strains isolated from retail meats showed an origin-specific composition and contained virulence and antimicrobial resistance genes similar to those present in human clinical isolates.

## Introduction

The bacterium *Staphylococcus aureus* can incite life-threatening infections in both humans and animals (Tong et al., 2015). Although nosocomial infections are common, the acquisition of methicillin-resistant *S. aureus* (MRSA) from communities and livestock is also responsible for clinical cases and the spread of antimicrobial resistant (AMR) strains (Miller, 2010; Mediavilla et al., 2012). The virulence factors, exoenzymes, and toxins produced by *S. aureus* genomes are responsible for pathogenesis (Tam and Torres, 2019). Staphylococcal food poisoning (SFP) related to consumption of contaminated food is a major problem worldwide, and staphylococcal enterotoxins are responsible for clinical symptoms (Hennekinne et al., 2012; Lin et al., 2016). In the United States, *S. aureus* remains responsible for about 241,148 annual cases of domestically acquired foodborne illness (Hedberg, 2011), which is typified by nausea and vomiting.

Both humans and animals play an important role in food product contamination during preparation and storage (Lozano et al., 2016), and food handlers are regarded as a major factor in contamination (Castro et al., 2016). A high prevalence of *S. aureus* contamination in retail meat and dairy products has been reported (Kitai et al., 2005; Abdalrahman and Fakhr, 2015; Abdalrahman et al., 2015b; Ge et al., 2017), and the most common clonal complexes were CC5 (chicken) and CC398 (turkey) (Waters et al., 2011; Thapaliya et al., 2017). A high incidence of toxin genes in food isolates indicates the potential virulence of these *S. aureus* strains (Abdalrahman and Fakhr, 2015; Abdalrahman et al., 2015a; Song et al., 2015; Ge et al., 2017; Gaerste-Díaz et al., 2018). Previous studies from our laboratory showed up to 37% staphylococcal contamination in retail chicken liver and gizzards (Abdalrahman and Fakhr, 2015) and 57% in poultry products (Abdalrahman et al., 2015a). Approximately 80% of retail beef liver samples, 50% of beef cuts, and 43% of pork samples were contaminated with *S. aureus* (Abdalrahman et al., 2015b). A high prevalence of staphylococcal toxin genes (e.g., *seg, sei, lukE-lukD, hla*, and *hld*) was reported in these strains (Abdalrahman and Fakhr, 2015; Abdalrahman et al., 2015a,b). Furthermore, *spa* typing of selected strains from retail chicken, turkey, chicken liver, and gizzards indicated a human origin (Abdalrahman and Fakhr, 2015; Abdalrahman et al., 2015a).

Multidrug resistant *S. aureus* strains, including MRSA, are prevalent in food products (Abdalrahman et al., 2015a; Ge et al., 2017; Sergelidis and Angelidis, 2017), especially in retail turkey meats (Abdalrahman et al., 2015a; Thapaliya et al., 2017). Livestock-associated MRSA can also cause clinical infections in humans (Larsen et al., 2017), and *S. aureus* strains in food products are often resistant to one or more antibiotics (Abdalrahman and Fakhr, 2015; Abdalrahman et al., 2015a,b; Jamali et al., 2015; Al-Ashmawy et al., 2016; Ge et al., 2017; Wu et al., 2018). *S. aureus* strains are generally screened for MRSA by PCR using *mecA* and *mecC*, and the recently characterized *mecB* may also play a role in methicillin resistance (Becker et al., 2018; Carretto et al., 2018). Recently, vancomycin-resistant *S. aureus* (VRSA) has become prevalent in retail meat products (Abdalrahman and Fakhr, 2015; Abdalrahman et al., 2015a,b). VRSA, VISA (intermediate resistance to vancomycin), and phenotypic MRSA strains (resistant to oxacillin and cefoxitin) have also been reported in retail meat and food products (Abdalrahman and Fakhr, 2015; Abdalrahman et al., 2015a,b). MRSA that lack *mecA* have been recovered from clinical specimens (Elhassan et al., 2015). In *S. aureus*, plasmids play important roles in the transfer and acquisition of antibiotic resistance genes (Haaber et al., 2017; Bukowski et al., 2019), which are often encoded by transposons, integrative conjugative elements (ICEs), staphylococcal chromosome cassettes (SCCs), and SaPI (Haaber et al., 2017). Furthermore, phage-related sequences in *S. aureus* may also encode genes for antimicrobial resistance (Baba et al., 2002, 2008; Xia and Wolz, 2014).

Whole genome sequencing (WGS) of *S. aureus* has focused primarily on clinical isolates (Baba et al., 2008; Shajari et al., 2017; Dweba et al., 2018), including MRSA (Dweba et al., 2018; Turner et al., 2019), and VRSA (Shajari et al., 2017). WGS has facilitated comparative analyses of *S. aureus* virulence factors, AMR, and pathogenicity islands (Baba et al., 2002, 2008; Nguyen et al., 2015; Bosi et al., 2016; Planet et al., 2017; You et al., 2018). A comparative study of 64 *S. aureus* clinical isolates revealed a conserved core genome of 1441 genes (Bosi et al., 2016). Furthermore, *S. aureus* pathogenicity islands (νSaα, νSaβ, and SaPI) encode exotoxin, lipoprotein, serine protease, and enterotoxin genes (Baba et al., 2002, 2008; Tam and Torres, 2019). Differences in the genomic composition of *S. aureus* pathogenicity islands and the type VII secretion system were also reported (Baba et al., 2002, 2008; Warne et al., 2016) and may contribute to differential pathogenicity.

Many studies describe the isolation and characterization of *S. aureus* strains from retail food products. However, few studies have used WGS to characterize strains from food products (Mossong et al., 2015; Ge et al., 2017; Sivaraman et al., 2017), and most of these focused on MRSA or strains related to food poisoning outbreaks (Mossong et al., 2015; Ge et al., 2017; Sivaraman et al., 2017). To our knowledge, WGS comparisons are lacking for *S. aureus* isolated from retail meats. We previously isolated multiple *S. aureus* strains from retail meat products and screened them for virulence and antimicrobial susceptibility (Abdalrahman and Fakhr, 2015; Abdalrahman et al., 2015a,b). The aims of this study were to sequence the whole genome of a selected set of plasmid-bearing *S. aureus* strains previously isolated from various retail meats, and to perform comparative genomics analysis to explore their potential virulence, AMR, and origin.

## Materials and Methods

### Bacterial Cultures and DNA Isolation

The 10 *S. aureus* strains used in this study were previously isolated from retail beef (*n* = 3), chicken (*n* = 4), turkey (*n* = 2), and pork (*n* = 1); all strains contained plasmid DNA based on alkaline lysis and PFGE (Table 1; Abdalrahman and Fakhr, 2015; Abdalrahman et al., 2015a,b). *S. aureus* strains were selected to represent various retail meat sources (beef, beef liver, chicken, chicken liver, chicken gizzard, pork, and turkey), different plasmid sizes previously determined by PFGE, and various plasmid *rep* types (Neyaz, 2019; Neyaz et al., 2020). DNA isolation and sequencing were carried out as described previously with minor modifications (Marasini and Fakhr, 2016a,b,c, 2017a,b; Neyaz et al., 2019). Briefly, *S. aureus* cells were grown in tryptic soy agar (TSA) at 37°C for 16–24 h and then used for genomic and plasmid DNA isolation. The DNeasy Blood and Tissue kit (QIAGEN Inc., Valencia, CA, United States) was used for genomic DNA isolation. The Qubit dsDNA HS Assay kit (Life Technologies, Carlsbad, CA, United States) was used for DNA quantification with a Qubit 2.0 fluorimeter.

**TABLE 1:** Origin and sequence characteristics of *S. aureus* strains used in this study.

***S. aureus* strain**	**Source**	**Chromosome**						**Plasmids**						
		**Size (bp)**	**No of contigs**	**Coverage (Avg.)**	**G+C**	**ORF**	**Accession no.**	**Name**	**Size (bp)**	**No of contigs**	**Coverage (Avg.)**	**G+C**	**ORF**	**Accession no.**
B1-4A	Beef	2,745,835	1	269	32.81	2587	CP042048	pSALNB2.8	2,777	1	18191.55	30.28	3	CP042049
								pSALNB22	22,915	1	161.67	34.08	30	CP042050
								pSALNB86	86,501	30	308.76	33.96	138	CP042051-CP042080
B2-7A	Beef liver	2,780,737	1	307.41	32.89	2603	CP042046	pSALNBL75	75,938	1	408.07	30.24	85	CP042047
B2-15A	Chicken liver	2,654,842	1	214.76	32.26	2447	CP042043	pSALNCL17	17,035	1	1806.38	28.11	28	CP042045
								pSALNCL1.4	1,435	1	28028.31	33.94	1	CP042044
B3-4A	Beef liver	2,747,092	1	119.61	32.88	2578	CP042008	pSALNBL2.8	2,899	1	4920.18	31.08	3	CP042009
								pSALNBL118	118,216	33	162.03	34.91	180	CP042010- CP042042
B3-14B	Turkey	2,819,586	1	161.03	33.07	2690	CP042003	pSALNT46	46,487	1	569.55	29.06	64	CP042004
								pSALNT16	16,596	1	514.52	28.47	26	CP042005
								pSALNT4.9	4,979	1	2655.8	30.23	6	CP042006
								pSALNT2.2	2,232	1	4890.27	32.35	6	CP042007
B3-17D	Chicken	2,840,146	1	242.54	32.85	2704	CP042157	pSALNC17	17,383	1	2436.25	29	23	CP042158
								pSALNC1.6	1,686	1	35691.46	35.5	2	CP042159
								pSALNC1.4-1	1,435	1	42493.55	34.08	1	CP042160
B4-59C	Chicken	2,781,607	1	204.33	32.82	2618	CP042153	pSALNC14	14,156	1	1043.61	29.39	24	CP042154
								pSALNC2.8	2,880	1	648.81	26.46	5	CP042155
								pSALNC1.4-2	1,435	1	6727.37	33.49	1	CP042156
B6-55A	Turkey	2,765,700	1	209.32	32.88	2633	CP042110	pSALNT20	20,895	1	185.34	36.19	28	CP042111
								pSALNT106	106,784	40	168.62	33.46	184	CP042112-CP042152
B8-13D	Chicken gizzard	2,807,514	1	169.45	32.86	2657	CP042107	pSALNCG17	17,383	1	2525.17	28.95	24	CP042108
								pSALNCG1.5	1,562	1	42959.6	33.99	2	CP042109
B9-22D	Pork	2,703,684	1	105.15	32.84	2505	CP042081	pSALNP2.8	2,806	1	7018.71	32.04	5	CP042082
								pSALNP9	9,395	1	1073.42	32.35	8	CP042083
								pSALNP58	58,407	23	58.14	29.84	77	CP042084-CP042106

### Library Preparation, Sequencing and Assembly

Sequencing libraries of genomic DNA were prepared and normalized with the Nextera XT DNA Library Preparation Kit (Illumina Inc., San Diego, CA, United States) as recommended by the manufacturer. Sequencing of prepared libraries was conducted in the Illumina MiSeq platform using the Illumina MiSeq V2 Reagent kit and 2 × 250 cycles. Sequence assembly was conducted using the CLC Genomic Workbench v. 7.0 and the microbial genome finishing module. Identification, clustering and segregation of plasmid sequences from chromosomal sequences was performed with plasmidSPAdes (Antipov et al., 2016) and the PHASTER web server (Arndt et al., 2016). Several contigs were joined manually by consulting reference sequences. Assembled genomic and plasmid sequences were deposited in GenBank as listed in Table 1. Sequences were annotated using the NCBI Prokaryotic Genome Annotation pipeline. RAST^1^ (Overbeek et al., 2014) and PATRIC v. 3.5.39^2^ (Wattam et al., 2014) tools were used to annotate whole genomic sequences of *S. aureus* for comparative genomic analysis.

### Multilocus Sequence and spa Typing

Assembled FASTA files of all sequenced *S. aureus* strains were analyzed. Multilocus sequence typing (MLST) and single locus typing (spa typing) of genomic sequences were conducted using MLST 2.0^3^ (Larsen et al., 2012) and spaTyper 1.0^4^ (Bartels et al., 2014), respectively.

### Comparative Genomic Analysis

Genomic comparisons among *S. aureus* strains were obtained by BLASTn analysis^5^. Nucleotide difference (ND) trees were created using NDtree-1.2 (Joensen et al., 2014; Kaas et al., 2014; Leekitcharoenphon et al., 2014) and FASTA files of the 10 *S. aureus* strains (this study) and the following reference strains from GenBank: *S. aureus* MRSA252 (accession no. BX571856.1), Mu50 (NC_002758.2), JH1 (CP000736.1), MW2 (NC_003923.1), COL (NC_002951.2), and N315 (NC_002745.2). GenBank files (.gbk files, RAST-annotated) of chromosomal and plasmid sequences were used for pangenome analysis in the GView Server^6^. Fifteen genomic sequences, including five from GenBank, were used for pangenome analysis. Similarly, pangenome analysis was performed for small plasmids (<5 kb), whereas the analysis on larger plasmids (>5 kb) was performed using RAST-annotated sequences in the GView Server. Values for percent identity cutoff (90), alignment length cutoff (100), and *e*-values (<1e^–10^) were used for BLASTn analysis in the GView Server. Genomic features of sequences were illustrated using PATRIC (3.5.39), the GView Server (display genomic feature tool), and CLC genomic workbench v. 12 (QIAGEN Bioinformatics). Genome tree report (based on genomic blast) of *S. aureus* strains (10453 genomes) from NCBI^7^ was obtained (retrieved on August 07, 2020) and phylogenetic trees from genome tree report were further analyzed in NCBI Genome workbench (version 3.4.1)^8^.

### Identification of Virulence Factors and AMR Genes

Assembled FASTA files of whole genomic sequences derived from the CLC workbench were used for virulence gene prediction using VFanalyzer^9^ (Liu et al., 2019), and the results were tallied with RAST- and PATRIC-annotated genomic features. Curated *S. aureus* virulence gene sequences from VFDB^10^ (Liu et al., 2019) were manually blasted against whole genome sequences of all 10 *S. aureus* strains. RAST- and PATRIC-annotated virulence genes that were identified in the virulence, defense and diseases subsystems and stress response genes (stress response subsystem) were listed and compared.

Raw FASTA files or assembled contigs of whole genome sequences were used in RGI^11^, ResFinder 3.1^12^ (Zankari et al., 2012), and CARD analysis (PATRIC annotation pipeline) for the prediction of AMR genes. Island viewer 4^13^ (Bertelli et al., 2017) was used for the identification and visualization of genomic islands. Genomic arrangements in νSaα, νSaβ, and SaPI genomic islands and the Type VII secretion system were illustrated using genomic features from Seed Viewer (RAST)^14^. *S. aureus* NCTC8325 was used as a reference strain for studying the Type VII secretion system.

### Phylogenetic Analysis Among Virulence Genes

Protein sequences for *clfA, fnbA*, and *spa* genes were downloaded from the RAST server. Protein sequence alignments were conducted with MUSCLE (Edgar, 2004)^15^ and analyses for maximum likelihood were conducted in Mega X (Kumar et al., 2018) with a bootstrap value of 100.

## Results

### Features of Genomic and Plasmid DNA Sequences

Details for the ten sequenced *S. aureus* strains including size, contig number, and G+C ratio are shown in Table 1. Chromosomes ranged from 2,654,842 to 2,807,514 bp, and the average G+C ratio was 32.26–33.07. Twenty-five plasmids were detected and sequenced from the 10 *S. aureus* strains, and all harbored one or more plasmids (Table 1). Plasmids ranged from 1.4 to 118 kb with a mean G+C ratio of 26.46–36.19. The mean ORF length ranged from 2447 to 2704 bp. Between 1 and 6 ORFs were identified in small plasmids (<5 kb), and 6–184 ORFs were present in larger plasmids (>5 kb).

### Comparative Genomics

**FIGURE 1:**
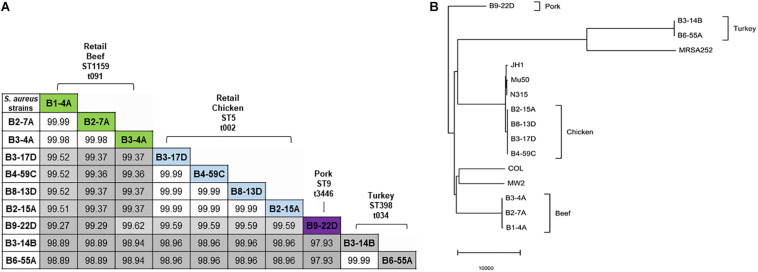
**(A)** Nucleotide sequence similarity in 10 *S. aureus* genomes. Sequence similarity values (%) are highlighted in gray (lowest value) to white (highest value). MLST types are indicated with prefix ST, and spa types with prefix t. **(B)** Nucleotide difference tree generated with *S. aureus* genomic sequences and NDtree v. 1.2.

**FIGURE 2:**
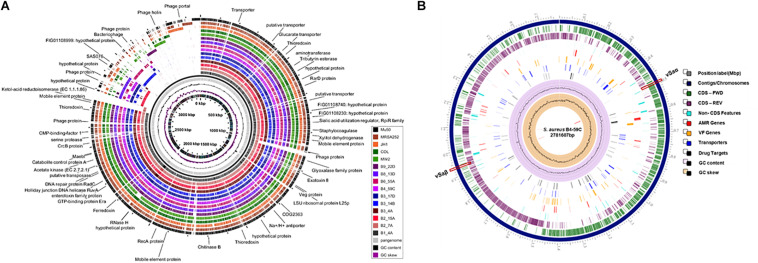
**(A)** Pangenome analysis of the 10 *S. aureus* strains described in this study and reference strains Mu50, MW2, JH1, MRSA252, and COL. Each circle depicts the sequence of a single strain. The pangenome (gray circle), GC content and GC skew are shown in the center. **(B)** Characteristics of the *S. aureus* B4-59C genome using PATRIC. AMR, antimicrobial resistance; VF, virulence factor.

Sequenced genomes from the same retail meat product showed greater similarity (%) to each other than sequences from different meats (Figure 1A). Sequence similarity values obtained from BLASTn analysis ranged from 97.93 to 99.99% (Figure 1A). Despite their variable origin (retail chicken, chicken liver, and chicken gizzard), all chicken strains shared similar MLST and spa types (ST5, t002). Similar results were found among beef (ST1159, t091), pork (ST9, t3446), and turkey isolates (ST398, t034). Nucleotide difference (ND) trees were created from genomic sequences, and NDtree v. 1.2 revealed clusters specific for meat sources (Figure 1B). Genome tree report (Supplementary Figure S1) showed that retail beef strains from our laboratory clustered together and most of the strains in neighboring clusters were clinical isolates from human host (Supplementary Figure S2 and Supplementary Table S1). Only two strain G08M and G11F in the neighboring clusters were from cattle (mastitis) which were isolated from milk. All retail chicken isolates from our study clustered with *S. aureus* strains mainly from broiler chicken (Supplementary Figure S3 and Supplementary Table S2). Strain B4-59C was found in the same cluster with *S. aureus* ED98 which has been studied in a previous report (Lowder et al., 2009). Blast analysis showed 99.98% similarity between genomic sequences of B4-59C and ED98 (Supplementary Figure S3). Retail pork isolate B9-22D was found to be clustered with mainly pork and cow isolates in Genome Tree report (Supplementary Figure S4 and Supplementary Table S3). Neighboring clusters included strains mainly from poultry and livestock. Likewise, most strains found in cluster with *S. aureus* strain B3-14B and B6-55A (retail turkey isolates) were from turkey source (Supplementary Figure S5 and Supplementary Table S4). Sequences were annotated and then analyzed for virulence, AMR and stress response genes (Figure 2B). Pangenome analysis showed differences between the *S. aureus* genomes from retail meats and genomes from reference clinical isolates (Figure 2A), most notably in hypothetical proteins, mobile elements and phage proteins (Supplementary Table S5).

### Virulence Factors

**FIGURE 3:**
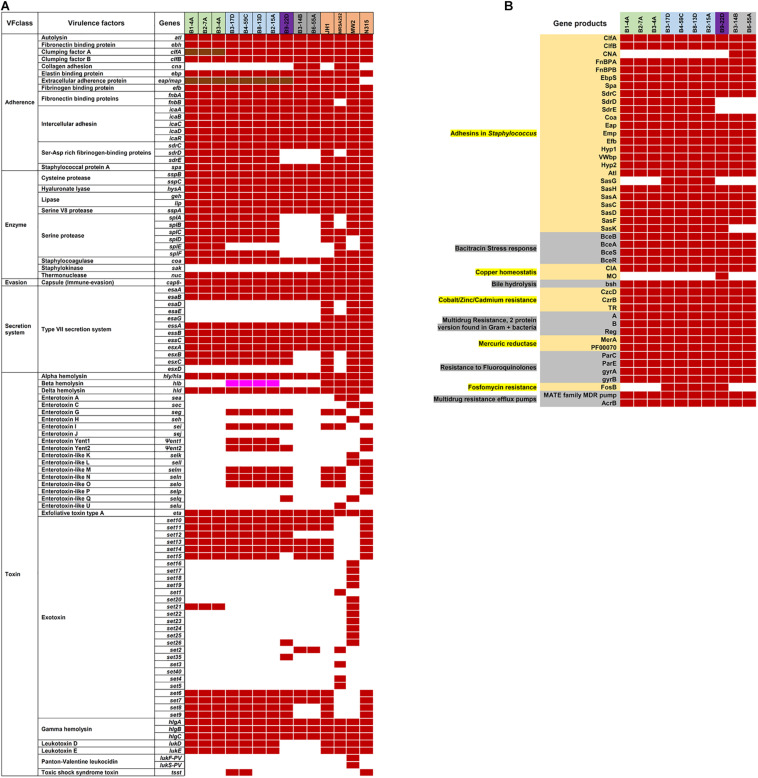
**(A)** Virulence genes in *S. aureus* strains are indicated by the following colors: red, present using VFanalyzer; brown, present in RAST-annotated genomes; white, absent; and pink, truncated. **(B)** RAST-annotated genes identified in subsystems of virulence, defense, and disease. Strain names in **(A,B)** are highlighted based on source as follows: green, retail beef; blue, retail chicken; purple, pork; and gray, turkey.

**FIGURE 4:**
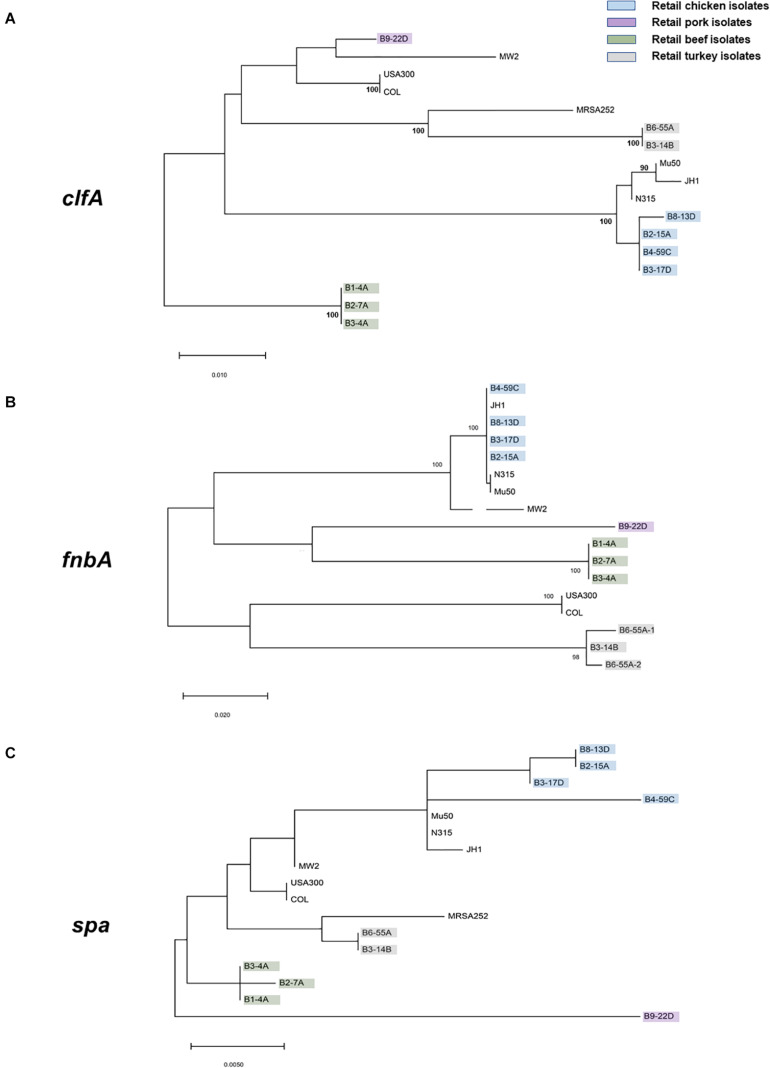
Maximum likelihood trees (bootstrap values of 100) derived from the predicted protein sequences of **(A)** *clfA*, **(B)** *fnbA*, and **(C)** *spa* genes. *S. aureus* strains characterized in this study are highlighted with the following colors: green, retail beef; blue, retail chicken; purple, pork; and gray, turkey. All remaining isolates are clinical reference strains.

Virulence and toxin genes were predicted and identified using VFanalyzer and RAST annotation, and the resulting patterns reflected the meat origin of the isolates (Figures 3A,B and Supplementary Table S6). Among genes related to adherence, differences were seen for collagen adhesion (*cna*), *sdrD*, *and sdrE*. The *sdrD* and *sdrE* genes were harbored by both beef and chicken isolates, whereas *cna* was only present in turkey isolates. Genes encoding *clfA* and *eap/map* were present in all RAST-annotated sequences but were not observed with VFanalyzer. Comparative analysis of protein sequences derived from *cflA*, *fnbA*, and *spa* genes revealed differences that were inferred from maximum likelihood analysis (Figures 4A–C). *spa* gene has been annotated as pseudogene in NCBI Prokaryotic Genome Annotation Pipeline (PGAP) for all retail chicken isolates. Retail chicken isolates grouped with clinical reference strains; however, beef, pork and turkey isolates were genetically distinct from most clinical strains (Figures 4A–C). Comparative analysis of the genomic sequences on the individual gene level supports the results obtained from the nucleotide difference tree created using whole genome sequences. Other adherence-related genes, including *atl, ebh, clfB, ebp, efb, fnbB, icaA-D*, *icaR*, and *sdrC*, were present in all sequenced strains. Among the exoenzymes, serine protease genes (*splA, splB, splC, splD*, and *splF*) were present in beef and chicken isolates; however, *splE* was only identified in beef isolates. Genes for cysteine protease, staphylocoagulase, and thermonuclease were present in all strains, but staphylokinase was absent.

Genes encoding sphingomyelinase (Supplementary Table S6) and hemolysin (*hly/hla, hld*, and *hlgA-C*) were identified in all strains, and a truncated *hlb* was present in chicken isolates (RAST annotation). Exotoxin genes (*set6, set7 set10, set11, set13*, and *set14*) were found in all strains, and chicken and pork isolates harbored one or more enterotoxin genes such as *seg*, *sei*, *Ψent1/2 selm*, *seln*, *slo*, and *selq*. Leukotoxin genes *lukD* and *lukE* were present in beef and chicken isolates, but absent in pork and turkey isolates. *lukF-PV* and *lukS-PV* were identified in all 10 RAST-annotated sequences; however, BLASTp annotated these as LukG and LukH leukotoxins in retail beef, chicken, and pork isolates. Isolates from retail turkey contained genes for β-pore-forming cytolysin and leucocidin S.

**FIGURE 5:**
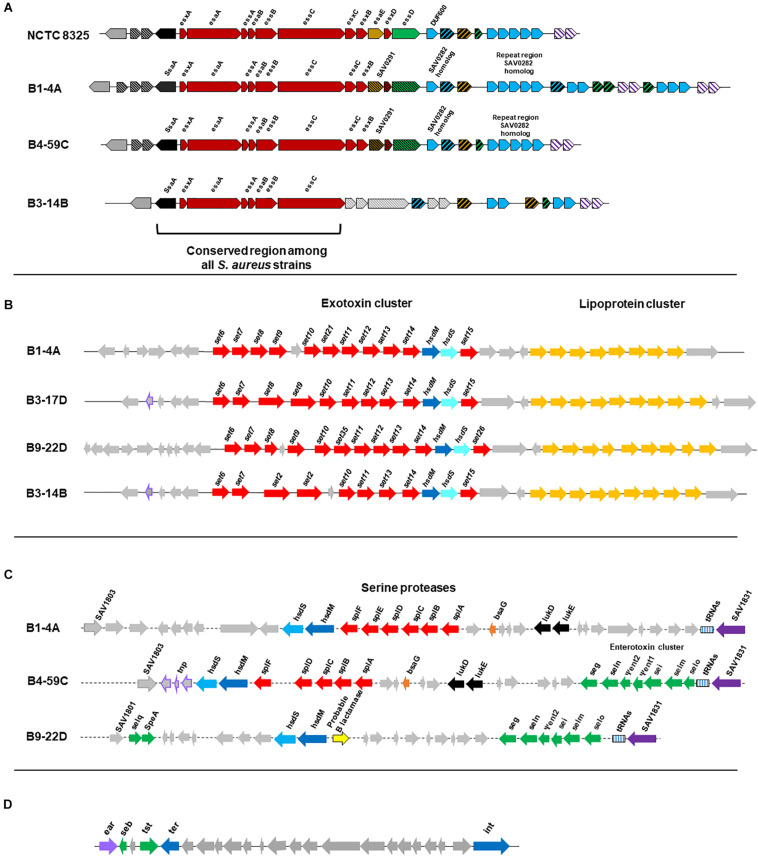
**(A)** Organization of gene clusters encoding the Type VII secretion system in *S. aureus* strains according to RAST annotation. The functional map for reference strain *S. aureus* NCTC8325 was adapted from a previous study (Warne et al., 2016). Cross-hatched and gray arrows indicate proteins with unknown functions. *S. aureus* strain B1-4A is a beef isolate that shares similarity with beef strains B2-7A and B3-4A, and the chicken isolate B4-59C is similar to the other three chicken strains (B3-17D, B8-13D, and B2-15A) and the pork isolate B9-22D. The map for the turkey isolate B3-14B is shown and shares similarity with B6-55A, which is also from turkey. **(B)** Organization of the νSaα genomic island and exotoxin cluster (*set* genes) in *S. aureus* strains. Genes were predicted from VFanalyzer and drawn to scale based on the genomic arrangement in RAST seed viewer. Arrows represent the following: red, coding sequence (CDS) for exotoxins; yellow, CDS for tandem lipoproteins; purple boundary, transposase; and gray, hypothetical proteins with unknown functions. *S. aureus* strains include representative isolates from beef (B1-4A), chicken (B3-17D), pork (B9-22D), and turkey (B3-14B). **(C)** νSaβ genomic island with serine proteases and enterotoxin gene cluster (*seg, sei, Ψent1/2, selm, seln, selo, selq*) in *S. aureus* strains. Genes were predicted from VFanalyzer and drawn to scale using the arrangement observed with the RAST seed viewer. Arrows represent the following: red, CDS for serine proteases; green, CDS for enterotoxins; purple, putative transposase; and gray, hypothetical proteins. *S. aureus* strains include representative isolates from beef (B1-4A), chicken (B4-59C), and pork (B9-22D). **(D)** *S. aureus* pathogenicity island (SaPI) carrying the gene for toxic shock syndrome toxin-1 and *seb* in *S. aureus* strain B4-59C. Genes were predicted from VFanalyzer and drawn to scale using the genomic arrangement observed with RAST seed viewer.

All 10 genomes contained genes related to the Type VII secretion system (e.g., *ssaA*, *esxA, esaA, esaB*, and *essA-C*). Beef strains also contained *esaC* and *esxB* and higher numbers of repeats than reference strain *S. aureus* NCTC8325 (Figure 5A). Although chicken and pork isolates shared similarity with reference strain NCTC8325, they contained proteins with unidentified functions instead of *esaE, esxD*, or *essD* (Figure 5A). Retail turkey isolates lacked *esxC, esxB* and the SAV0291 homolog, and exhibited differences in the repeat regions.

### Genomic Islands

The genomic island νSaα, containing exotoxin *set* genes and a tandem lipoprotein cluster, was present in all genomes; however, differences were observed in *set* gene arrangement (Figure 5B). The νSaβ island, which encodes the enterotoxin gene cluster (*egc*), was present in chicken and pork isolates (Figure 5C). Beef isolates lacked an enterotoxin gene cluster but possess a serine protease cluster. Interestingly, the toxic shock syndrome toxin gene (*tsst*) was encoded by the SaPI genomic island in strains B4-59C (Figure 5D) and B3-17D.

### AMR and Stress Response Genes

**FIGURE 6:**
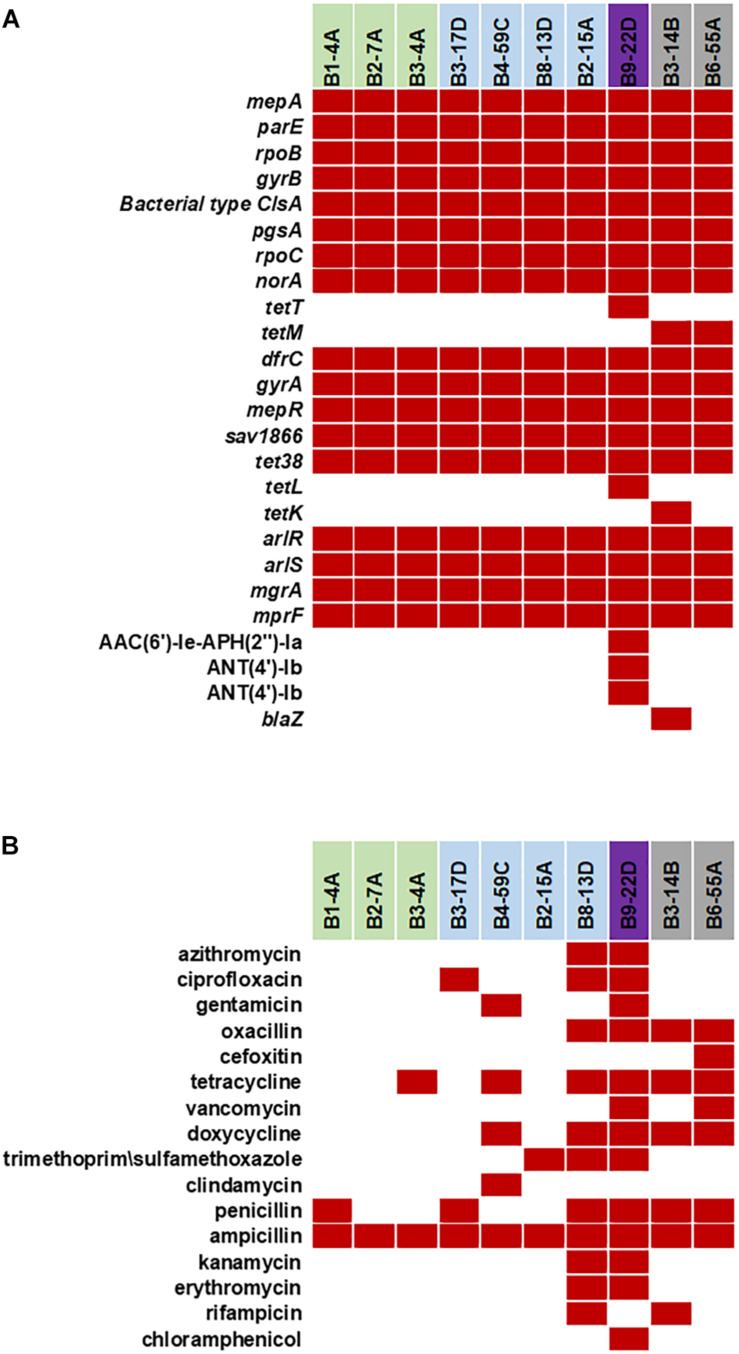
**(A)** Antimicrobial resistance genes in the 10 sequenced *S. aureus* strains. Red solid blocks indicate that a gene was present. **(B)** Antimicrobial resistance pattern in the 10 foodborne *S. aureus* strains. AMR screening was reported previously (Abdalrahman and Fakhr, 2015; Abdalrahman et al., 2015a,b) (Red block – resistant, white block – sensitive).

Various AMR genes were identified in the sequenced genomes (Figure 6A). The tetracycline resistance (Tet^*R*^) gene *tetM* was present in the chromosome of turkey isolates, and *tetK, tetL*, and *tetT* were plasmid-encoded. Beef and chicken isolates harbored similar AMR genes (Figure 6A). All strains encoded bacitracin stress response genes *bceA, bceB, bceR*, and *bceS* (Figure 3B). Genes related to multidrug resistance, fluoroquinolone resistance (*parC parE, gyrA gyrB*) and multidrug reistance efflux pumps (*acrB*, MATE family MDR pump) were present in all strains (Figure 3B). The fosfomycin-resistance gene *fosB* was present in chicken and pork isolates but absent in beef and turkey isolates (Figure 3B). All strains harbored genes related to cobalt/zinc/cadmium resistance (CzcD, CzrB, and TR) and the stress response subsystem (Table 2). Genes encoding nitric oxide reductase (EC1.7.99.7) and a quinol-dependent gene were only present in turkey isolates.

**TABLE 2:** Common stress response genes found in genomes of *S. aureus* strains (RAST subsystem – stress response).

**Sub-Class**	**Subsystem**	**Gene products**
Stress response: Electrophile toxicity	Bacillithiol synthesis	Glucosaminyl-malate:cysteine ligase
		N-acetylglucosaminyl-L-malate N-acetyl hydrolase
		UDP-N-acetylglucosamine:L-malate glycosyltransferase
Stress response: oxidative stress/undefined	Cluster containing Glutathione synthetase	16S rRNA (uracil(1498)-N(3))-methyltransferase (EC 2.1.1.193)
		Putative pre-16S rRNA nuclease YqgF
	CoA disulfide thiol-disulfide redox system	CoA-disulfide reductase (EC 1.8.1.14)
	Glutathione: Redox cycle	Glutathione peroxidase (EC 1.11.1.9) @ Thioredoxin peroxidase (EC 1.11.1.15)
	Hydroxy-fatty acid production as stress response	Oleate hydratase (EC 4.2.1.53)
	Protection from Reactive Oxygen Species	Catalase KatE (EC 1.11.1.6)
		Superoxide dismutase [Mn] / [Fe] (EC 1.15.1.1)
	Universal stress protein family	Universal stress protein family
	Repair of Iron Centers	Nitric-oxide reductase (EC 1.7.99.7), quinol-dependent
		Repair of Iron Centers di-iron protein
Stress response: Osmotic stress	Choline uptake and conversion to betaine clusters	Betaine aldehyde dehydrogenase (EC 1.2.1.8)
		Betaine/carnitine/choline transporter (BCCT) family
		Choline ABC transport system, ATP-binding protein OpuBA
		Choline ABC transport system, choline-binding protein OpuBC
		Choline ABC transport system, permease protein OpuBB
		Choline ABC transport system, permease protein OpuBD
		Choline dehydrogenase (EC 1.1.99.1)
		FIG009707: Betaine operon transcriptional regulator
		Glycine betaine ABC transport system, ATP-binding protein OpuAA (EC 3.6.3.32)
		Glycine betaine ABC transport system, permease protein OpuAB
		Glycine betaine ABC transport system, glycine betaine-binding protein OpuAC
		Glycine betaine transporter OpuD
	Osmoregulation	Glycerol uptake facilitator protein
Stress response: Heat/Cold shock	Cold shock proteins of CSP family	Cold shock protein of CSP family
	Heat shock dnaK gene cluster extended	16S rRNA (cytidine(1402)-2′-O)-methyltransferase (EC 2.1.1.198)
		16S rRNA (uracil(1498)-N(3))-methyltransferase (EC 2.1.1.193)
		Chaperone protein DnaJ
		Chaperone protein DnaK
		DNA replication initiation control protein YabA
		Phosphoesterase
		Heat shock protein GrpE
		Heat-inducible transcription repressor HrcA
		Nucleoside 5-triphosphatase RdgB (dHAPTP, dITP, XTP-specific) (EC 3.6.1.66)
		Oxygen-independent coproporphyrinogen-III oxidase-like protein YggW
		Ribosomal protein L11 methyltransferase
		tmRNA-binding protein SmpB
		Translation elongation factor LepA

*Retail beef isolates: B1-4A, B2-7A, and B3-4A; retail chicken isolates: B3-17D, B4-59C, B8-13D, and B2-15A; retail pork isolate: B9-22D; and retail turkey isolates: B3-14B and B6-55A. All genes were present among all *S. aureus* isolates except Nitric-oxide reductase (EC 1.7.99.7), quinolo-dependent which was found absent among *S. aureus* isolates from retail beef, retail chicken and retail pork.*

### Plasmids in *S. aureus* Strains

The plasmids sequenced in this study were previously characterized by S1 nuclease PFGE (Marasini and Fakhr, 2014; Cornell et al., 2018). A total of 25 plasmids were sequenced including 10 small (<5 kb) plasmids (Tables 1, 3). Five small plasmids were <2 kb (1435–1686 bp) and harbored genes annotated as hypothetical proteins (Supplementary Table S7). These plasmids were identified as poultry-specific pAvY-B1 (Bukowski et al., 2019) (pSALNCL1.4, pSALNC1.4-1, pSALNC1.4-2, and pSALNCG1.5) and pAvY (Bukowski et al., 2019) plasmids (pSALNC1.6), which were reported previously in a recent report (Bukowski et al., 2019; Table 3). The remaining five small plasmids harbored replication and hypothetical proteins (Supplementary Figures S6A,B and Supplementary Table S7). pSALNB2.8 and pSALNBL2.8 were related to pNVH01 (AJ512814.1) from horses (Bjorland et al., 2003), pSALNT2.2 was similar to pSR04 (CP019567.1) from humans, and pSALNC2.8 was identified as a pAVX (Bukowski et al., 2019) plasmid (Table 3). pSALNBL2.8 and pSALNB2.8 harbored the small multidrug resistance (SMR) family protein (Supplementary Figure S6A and Supplementary Table S7), and pSALNC2.8 contained a staphopain A precursor gene (Supplementary Figure S6B). Interestingly, pSALNP2.8, and pSALNP9 did not show strong identity to other plasmid sequences deposited in GenBank.

**TABLE 3:** Plasmids sequenced in this study.

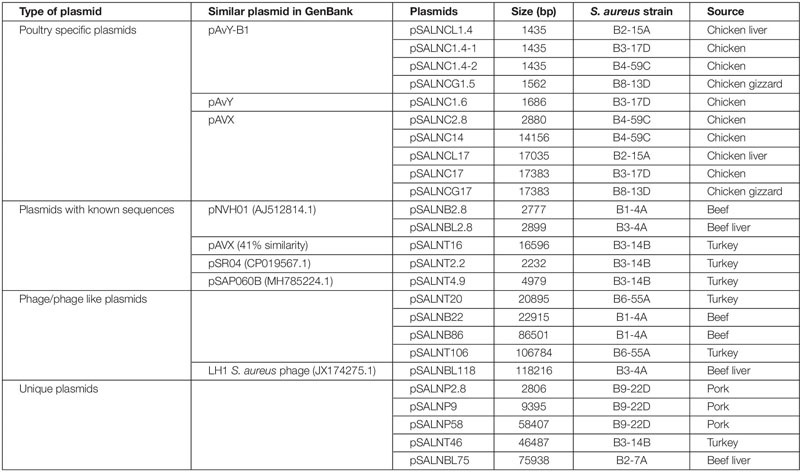

**FIGURE 7:**
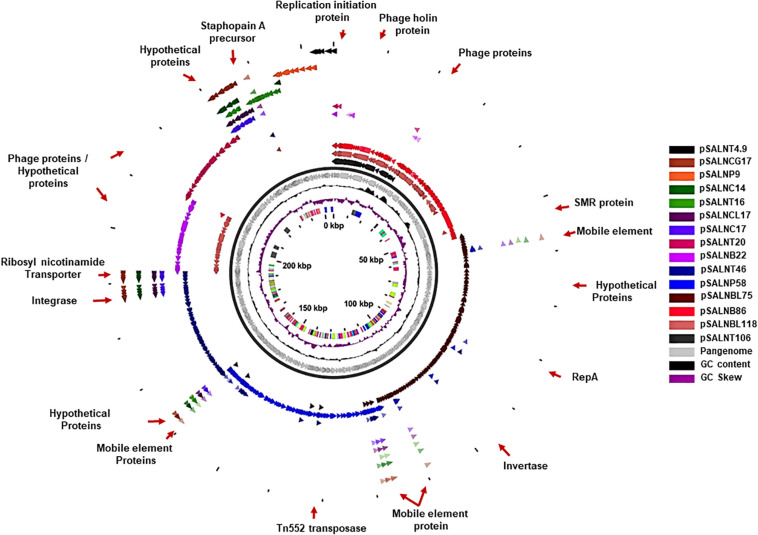
Pangenome of plasmids >5 kb in the *S. aureus* strains.

**FIGURE 8:**
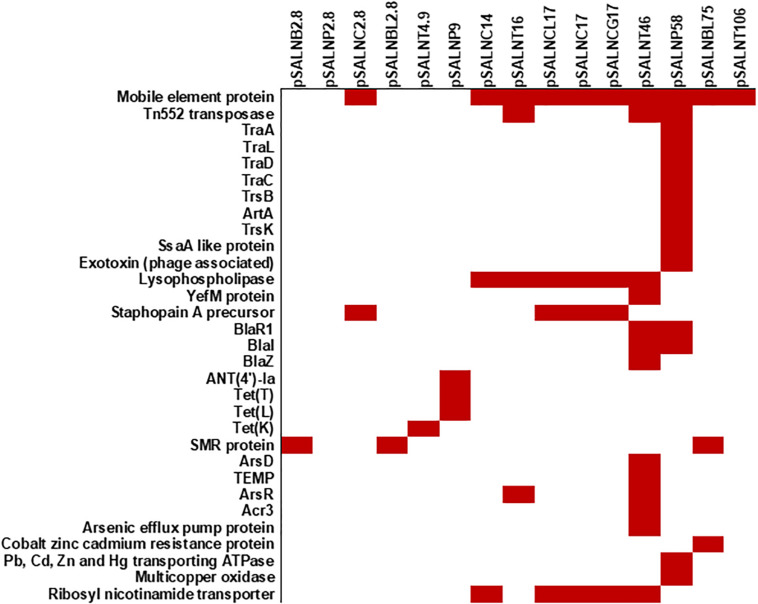
Plasmid-encoded genes potentially responsible for transfer, virulence, antimicrobial resistance, and heavy metal resistance in *S. aureus* strains. Solid red rectangles indicate that the gene was present. All gene products were annotated in RAST. Small (<5 kb) plasmids with hypothetical proteins (pSALNCL1.4, pSALNC1.4-1, pSALNC1.4-2, pSALNCG1.5, pSALNC1.6, and pSALNT2.2) and phage-like plasmids harboring only phage proteins (pSALNT20, pSALNB22, pSALNB86, and pSALNBL118) were excluded.

The larger plasmids (>5 kb) shared genes for mobile elements, hypothetical proteins, and integrase (Figures 7, 8 and Supplementary Table S8). Tet^*R*^ genes were identified in pSALNP9 [*tetL* and *tetT*] and pSALNT4.9 (*tetK*) (Supplementary Figures S7A,B). Moreover, pSALNT4.9 was related to pSAP060B (MH785224.1) with query cover 78% and 96.69% similarity; both plasmids contain Tet^*R*^ genes. Among the five plasmids ranging from 14,156 to 17,383 kb, the pAVX type plasmids (pSALNCL17, pSALNC17, pSALNC14, and pSALNCG17) were similar in genomic composition (Supplementary Table S8); however, pSALNT16 from turkey shared only 41% similarity with pAVX plasmids in GenBank. Three plasmids from retail chicken isolates, pSALNCL17, pSALNCG17 and pSALNC17, encoded staphopain A precursor gene (Supplementary Figures S8A,B). pSALNP58 shared 72% similarity with SAP068A (GQ900421.1) from hospital-acquired *S. aureus* strains. All remaining >5 kb plasmids encoded phage-related sequences; an example is pSALNBL118, which is 98% similar to the LH1 *S. aureus* phage (JX174275.1) sequence (Table 3).

pSALNT46 encoded β-lactamase (BlaZ family) and arsenate resistance genes (Supplementary Figure S9A). Similarly, *blaI, blaR*, and Tn552 transposase genes were encoded by pSALNT46 and pSALNP58. Plasmid pSALNP58 harbored heavy metal transporting ATPase, aminoglycoside N(6′)-acetyltransferase, and *traC/D/L* and *trsB*. The small multi-drug resistance (SMR) family protein gene was encoded by pSALNBL75 (Supplementary Figure S9B). The pSALNT20 sequence was comprised primarily of unique phage genes (Supplementary Figure S10B), and other phage-related genes were encoded by pSALNB22, pSALNB86, pSALNT106, and pSALNBL118 (Supplementary Figure S10 and Supplementary Table S8).

## Discussion

The *S. aureus* strains used in this study were previously isolated from retail meat products and characterized for enterotoxin genes and antimicrobial susceptibility (Abdalrahman and Fakhr, 2015; Abdalrahman et al., 2015a,b). Although the number of *S. aureus* strains used here was relatively small (*n* = 10), an origin-specific genomic composition for the strains was clearly observed. Genomic comparisons (Figure 1A) and clustering of *S. aureus* strains (Figure 1B) indicate that strains from the same meat origin (e.g., beef, chicken, turkey or pork) shared a similar genomic composition. Genome report tree showed the clustering of genomes of retail chicken isolate from our study with poultry isolates, retail pork isolate with pork isolates, and retail turkey isolates with turkey isolates from GenBank (Supplementary Figure S1–S5 and Supplementary Tables S2–S5). This support that these isolates originated from respective animal sources either due to systemic infection, colonization or during slaughtering and processing. Interestingly, retail beef isolates didn’t cluster with any other strains, but neighboring cluster mainly includes strains from human sources. Most of the whole genome sequenced S. *aureus* sequences in GenBank are from human sources. Hence, it is not surprising to see most of the strains from retail meat sources clustering with human strains. Meanwhile, previous reports from our laboratory had also showed that *S. aureus* strains mainly originated from retail beef (retail beef liver) were similar to *S. aureus* strains from human sources (Abdalrahman and Fakhr, 2015; Abdalrahman et al., 2015a,b).

NDtree constructed using our strains and few reference clinical strains has shown the proximity of retail chicken isolates with JH1, Mu50, and N315 clinical strains (Figure 1B). Retail chicken isolates and these clinical strains are also found in neighboring clusters in Genome report tree (Supplementary Figure S1). The arrangement of the νSaα genomic island in the four retail chicken isolates was similar to the Type I νSaα genomic island in clinical strain *S. aureus* N315 (Baba et al., 2002, 2008). Retail chicken isolates B3-17D and B4-59C harbored the toxic shock syndrome toxin gene (*tsst*), which was 100% similar to *tsst* in clinical strain N315. Interestingly, prior studies suggested a human origin for *S. aureus* isolated from retail chicken (Abdalrahman and Fakhr, 2015; Abdalrahman et al., 2015a). However, the origin of poultry ST5 clade has been suggested to be due to the recent human to poultry host jump with adaptation in poultry hosts (Lowder et al., 2009). The Genome tree report showed the clustering of WGS retail chicken isolates from our laboratory with genomes of *S. aureus* strains from chicken in GenBank (Supplementary Figure S3 and Supplementary Table S2). Strain B4-59C shared higher genomic similarity with strain ED98 (Supplementary Figure S3). Many functional genes, found in human clinical strains which play role in human host pathogenesis, had been documented to have mutation to create pseudogenes among poultry associated *S. aureus* strains (Lowder et al., 2009). Acquisition of novel mobile genetic element had also played major role for genetic diversification among poultry specific ST5 *S. aureus* strains. Meanwhile, enhanced resistance toward the chicken heterophils was found by poultry specific *S. aureus* ED98 (Lowder et al., 2009). Many genes including *spa* and *asp1* were found to be pseudogenes in annotation from PGAP for all our retail chicken isolates similar like *S. aureus* ED98. Presence of poultry specific plasmids (pAvY-B1, pAvY, pAVX) (Table 3) in retail chicken isolates from our study which supports their poultry associated origin. Although the presence of similar virulence genes is predicted in genomes of retail chicken isolates from this study with reference clinical strains, their virulence on chicken or human host have not been assayed which remains a limitation of this study and warrants for future investigation.

The pork isolate was assigned to ST9, a MLST group prevalent in pigs (Neela et al., 2009), which supports a livestock origin for pork isolate B9-22D. Many *S. aureus* strains found in cluster with B9-22D were isolated from pork skin and pig source (Supplementary Figure S4 and Supplementary Table S3). The turkey isolates were assigned to ST398, that also includes a dominant type of livestock-associated (LA) MSRA (Smith and Pearson, 2011) in CC398. Differences in virulence and AMR genes were previously observed in LA-MRSA (CC398) and community-associated (CA)-MRSA strains in a mouse model (Randad et al., 2019). Although ST398 MRSA had been well documented for its involvement in clinical cases in livestock and human, methicillin susceptible ST398 (MSSA) has also been reported as emerging public health threat (Valentin-Domelier et al., 2011). ST398 strains causing human infection are found not only as livestock associated but also found in livestock free environment (Diene et al., 2017). Previous studies had shown major factor influencing for pathogenesis in human hosts is the presence of prophages in *S. aureus* genome carrying virulence related genes (Diene et al., 2017; Kashif et al., 2019). Although, two retail turkey isolates used in this study (B3-14B and B6-55A) lack *mec* genes, phenotypic resistance toward oxacillin and cefoxitin was observed for these strains (Abdalrahman and Fakhr, 2015; Abdalrahman et al., 2015a,b). In this study, we observed that virulence factors in LA-CC398 *S. aureus* strains IHW398-1 and IHW398-2 (Randad et al., 2019) were similar to B3-14B and B6-55A. Presence of *tetM* gene, Tn*916* and lack of φ3 prophage indicate livestock origin of these strains. In genome report tree, both retail turkey isolates cluster with *S. aureus* strains (MRSA as well as MSSA) from turkey source (Supplementary Figure S5). Interestingly, strain B3-14B from our study is found highly similar to SA33924 strain that had been described as human adapted ST398 (blood stream infection isolate) in a previous report (Diene et al., 2017).

The *S. aureus* strains sequenced herein encoded virulence-related genes associated with adherence, enzyme secretion, immune system evasion, toxins, and leucocidins. Lipoproteins are known to function in immune system stimulation and invasion in human cells (Nguyen et al., 2015), which were also harbored by all our strains. Furthermore, the type VII secretion system genes *esxA, esaA, esaB*, and *essA-C* (Warne et al., 2016) were present in all strains. Meanwhile, these strains also lack many virulence related genes and phage proteins found in studied reference clinical strains (Supplementary Table S6). Presence of virulence related genes and AMR genes might play role in pathogenesis and adaptation in animals and human hosts. However, the virulence potential of these strains has not been assayed in this study which limits our assertion for their pathogenic nature in animals or human hosts. Enterotoxin genes were identified in retail chicken and pork strains (Figure 5C), which suggests a potential role in enterotoxin-mediated food poisoning.

The *S. aureus* strains in this study were resistant to antimicrobial compounds (Figure 6B; Abdalrahman and Fakhr, 2015; Abdalrahman et al., 2015a,b). Despite having *mecA, mecB* or *mecC* in their genome, all sequenced strains were resistant to ampicillin. Several strains (B8-13D, B9-22D, B3-14B, and B6-55A) found to be phenotypic MRSA and were resistant to oxacillin (Abdalrahman and Fakhr, 2015; Abdalrahman et al., 2015a,b). Antimicrobial resistance genes detected in plasmids sequenced in our study (Figures 6A,B) correlate with the phenotypic antibiotic resistance patterns seen in the *S. aureus* strains harboring them (Figure 6B). Ampicillin resistance might be partially mediated by pSALNT46 and pSALNP58, which contain Tn*552*-encoded *blaI/blaR* genes. The pork isolate B9-22D was kanamycin resistant, and the aminoglycoside 4′-nucleotidyltransferase gene borne on pSALNP9 might impart Km resistance. Strains B3-4A, B4-59C, B8-13D, B9-22D, B3-14B, and B6-55A were Tet^*R*^ and encode *tet*(38) in their genome (Figures 3B, 6A). The gene *tetM* is commonly encoded by Tn*916* and Tn*5801* in *S. aureus* from humans or by Tn*916* in animals (De Vries et al., 2009). The two turkey isolates harbor *tetM* in Tn*916*; furthermore, pSALNT4.9 and pSALNP9 plasmids in B9-22D and B3-14B were shown to encode *tetK, tetL*, and *tetT*. Plasmids pSALNB2.8 and pSALNBL75 from retail beef isolates harbored genes encoding SMR proteins; this is significant because SMR family proteins confer resistance to antispetics and quaternary ammonium products (Bay and Turner, 2009; Costa et al., 2013). The presence of genes on plasmids for arsenic efflux pump protein, cobalt zinc cadmium resistance protein, Pb, Cd, Zn, and Hg transporting ATPase, multicopper oxidase, SMR proteins might play possible roles in AMR and heavy metal resistance mechanisms of their hosts. It also emphasizes the role of plasmids in dissimination of resistance genes among *S. aureus* strains. The unique genomic composition of pSALNP2.8 and pSALNP9 plasmids when compared to all available plasmid sequences in GenBank warrants further studies in regards to plasmids from retail pork isolates. Phage or phage-like sequences are prevalent in *S. aureus* strains and are important for horizontal gene transfer (Goerke et al., 2009). Plasmids pSALNB22, pSALNT20, pSALNB86, pSALNBL75, pSALNT46, and pSAlNT106 contained phage-related proteins, and these plasmids might be important in the transmission of virulence factors.

Plasmids harbored by *S. aureus* are known to carry various genes responsible for its survival and adaptation. Lineage specific distribution of plasmids has been suggested in regards to the presence of resistance and virulence determinants (McCarthy and Lindsay, 2012). Plasmids in *S. aureus* have been categorized according to *rep* gene families (McCarthy and Lindsay, 2012) as well as the presence of functional genes (Bukowski et al., 2019). Poultry specific plasmids (Group I) (Bukowski et al., 2019) like pAvY-B1 (pSALNCL1.4, pSALNC1.4-1, pSALNC1.4-2, and pSALNCG1.5) and pAvY plasmids (pSALNC1.6) are cryptic plasmids. Likewise, another small plasmid, pSALNT2.2, from a retail turkey isolate bears only a gene coding for a replication protein. Beneficiary roles of these cryptic plasmids for the *S. aureus* host strains are yet to be identified. One study has hypothesized that cryptic plasmids serve as flexible vectors to acquire mobile genomic elements which leads to the origination of plasmids with known functional genes (Attéré et al., 2017). Staphopain A (Laarman et al., 2012) and lysophospholipase (Daugherty and Low, 1993) are well documented virulence factors which are found prevalent in poultry specific pAvX plasmids sequenced in our study. pSALNC2.8 shares 100% genomic similarity with pSALNC17 and pSALNCG17 and other pAvX plasmids available in GenBank despite the discrepencies on their sizes. Although, some plasmids (pSALNB2.8, pSALNP2.8, pSALNC2.8, and pSALNBL2.8) have similar size (∼2.8 kb), origin specific differences could be seen among these plasmids (Supplementary Table S3).

In summary, staphylococcal food poisoning (SFP) remains a major health problem, and outbreaks have been documented around the globe (Argudín et al., 2010; Kadariya et al., 2014; Johler et al., 2015; Mossong et al., 2015; Vitale et al., 2015; Stewart, 2017; Thapaliya et al., 2017). Contamination of retail food products with *S. aureus* and the presence of enterotoxins in foodborne strains has increased the potential for SFP outbreaks (Crago et al., 2012; Abdalrahman et al., 2015a,b; Jamali et al., 2015; Mossong et al., 2015; Al-Ashmawy et al., 2016; Ge et al., 2017). The 10 *S. aureus* strains isolated from retail meat products in this study contain various virulence factors and AMR genes. Sequencing more strains from various retail meat products will be ideal to represent the population of *S. aureus* circulating in retail meat. As concluding remarks, genomic analysis supported that all sequenced *S. aureus* strains from retail meat products in this study originated from respective animal sources. Source specific differences in virulence factors and genomic composition was found among the strains in comparative genomic analysis. As *S. aureus* strains with potential viurlence factors and stress response genes are retained in retail meat products even after processng and storage conditions, these strains might play role in possible food poisioning outbreaks and other clinical cases. Hence, enhancement in food safety measures for reduction of foodborne pathogens including *S. aureus* contamination is required. The pathogenicity of these strains studied in this study has not been tested but remains possible and warrants further investigation.

## Data Availability Statement

The datasets presented in this study can be found in online repositories. The names of the repository/repositories and accession number(s) can be found in the article/Supplementary Material.

## Author Contributions

MF: research design. AK and LN: experimental procedures, sequence assembly, and comparative genomics analysis. AK, LN, and MF: manuscript preparation. All authors contributed to the article and approved the submitted version.

## Conflict of Interest

The authors declare that the research was conducted in the absence of any commercial or financial relationships that could be construed as a potential conflict of interest.

## Footnotes

1 http://rast.theseed.org/FIG/rast.cgi

2 https://www.patricbrc.org

3 https://cge.cbs.dtu.dk/services/MLST/

4 https://cge.cbs.dtu.dk/services/spatyper/

5 https://blast.ncbi.nlm.nih.gov/Blast.cgi

6 https://server.gview.ca/

7 https://www.ncbi.nlm.nih.gov/genome/tree/154?

8 https://www.ncbi.nlm.nih.gov/tools/gbench/

9 http://www.mgc.ac.cn/cgi-bin/VFs/v5/main.cgi?func=VFanalyzer

10 http://www.mgc.ac.cn/VFs/

11 https://card.mcmaster.ca/analyze/rgi

12 https://cge.cbs.dtu.dk/services/ResFinder/

13 http://www.pathogenomics.sfu.ca/islandviewer/

14 http://rast.theseed.org/FIG/rast.cgi

15 https://www.ebi.ac.uk/Tools/msa/muscle/
