# Supplementary material for: Comparative Genomics of Plasmid-Bearing Staphylococcus aureus Strains Isolated From Various Retail Meats
Source: Front Microbiol. 2020 Oct 23;11:574923. doi: 10.3389/fmicb.2020.574923 (PMC7644949; doi:10.3389/fmicb.2020.574923)
Supplement: Supplementary file 2 [file Data_Sheet_2.pdf]

Supplementary Figures

Comparative genomics of plasmid-bearing *Staphylococcus aureus* strains isolated from various retail meats

Anand B. Karki[1#], Leena Neyaz[1#], and Mohamed K. Fakhr[1*]

[1]Department of Biological Science, The University of Tulsa, Tulsa, OK, 74104, USA

[#]These authors contributed equally to this work

Correspondence:

Mohamed K. Fakhr

[*]mohamed-fakhr@utulsa.edu

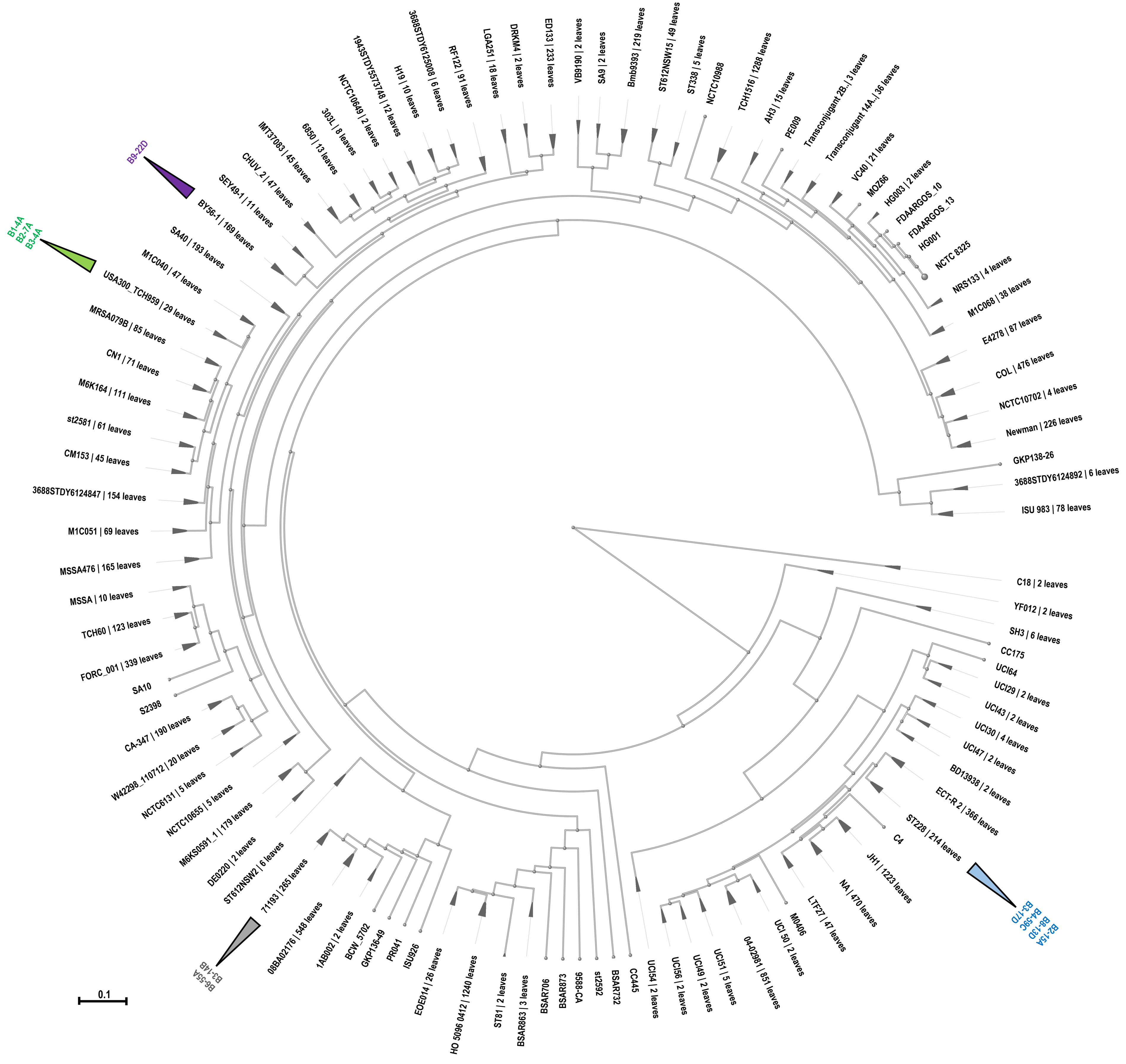

Fig. S1. Genome Report tree of *S. aureus* strains (**genomes = 10465**) retrieved from NCBI database on August 8th, 2020. Strains from our laboratory were indicated in the clusters with colorful arrows [Blue=chicken, green=retail beef isolates, purple= retail pork isolates, grey = retail turkey isolates

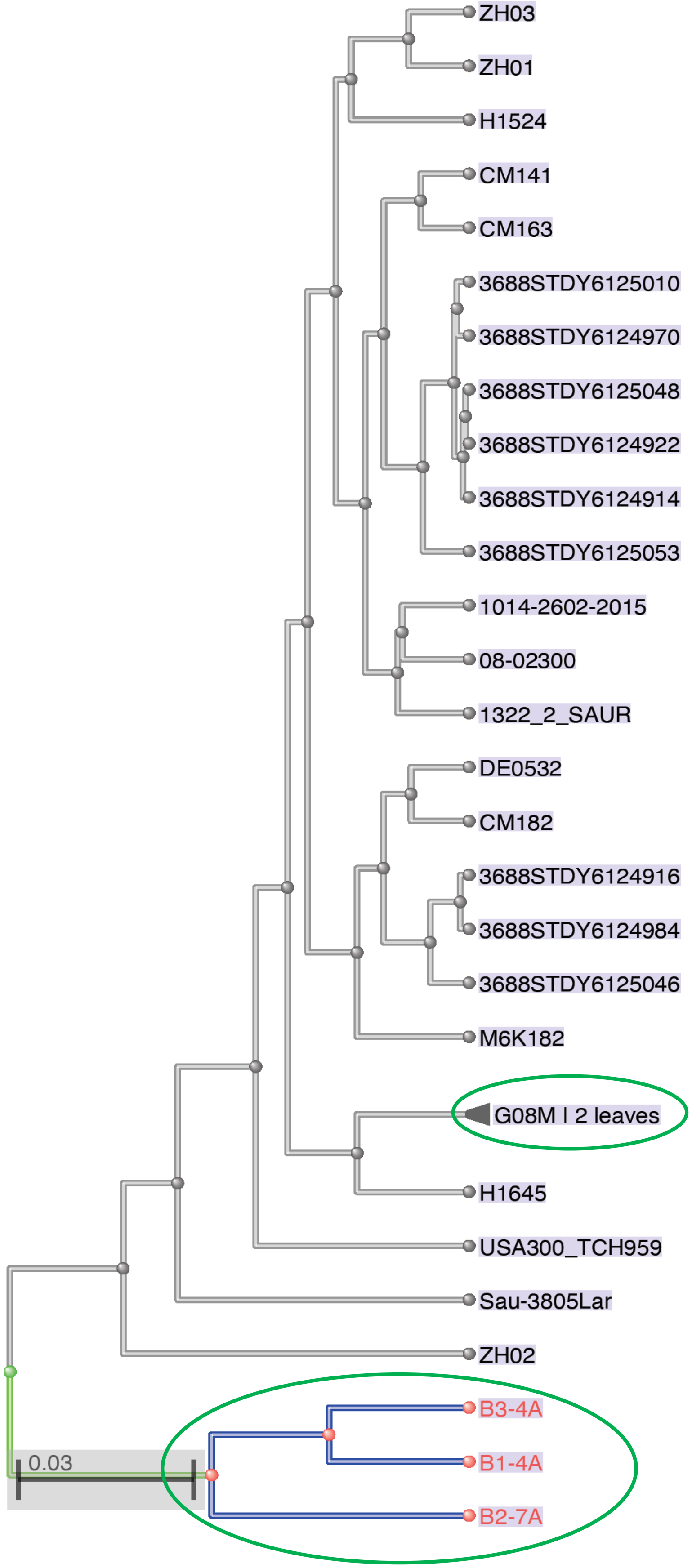

Fig. S2. Subtree of the phylogram (**Fig. S1**) that included retail beef isolates from our laboratory (strains name in red). Green circles indicate the strains from retail beef source or cattle source (clinical). Details of strains in this subtree is listed in **Table S2**.

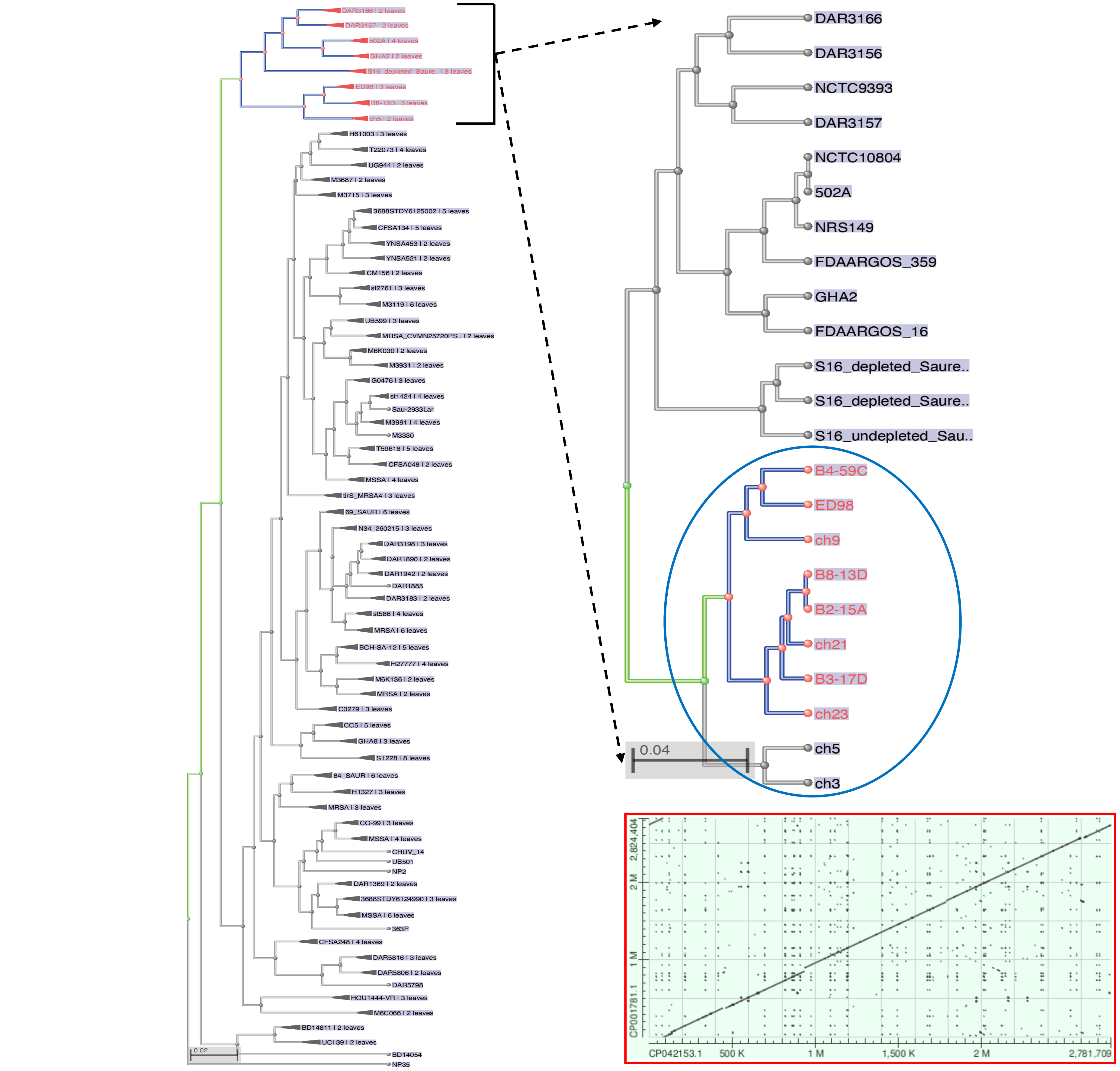

Fig. S3. Subtree of the phylogram (**Fig. S1**) that included retail chicken isolates from our laboratory (**B4-59C, B2-15A, and B3-17D**). Blue circle indicates the strains from chicken source. Details for the strains in the enlarged subset of subtree is listed in **Table S3**. Dot plot matrix (in red box) of *S. aureus* B4-59C vs. ED98 (BlastN).

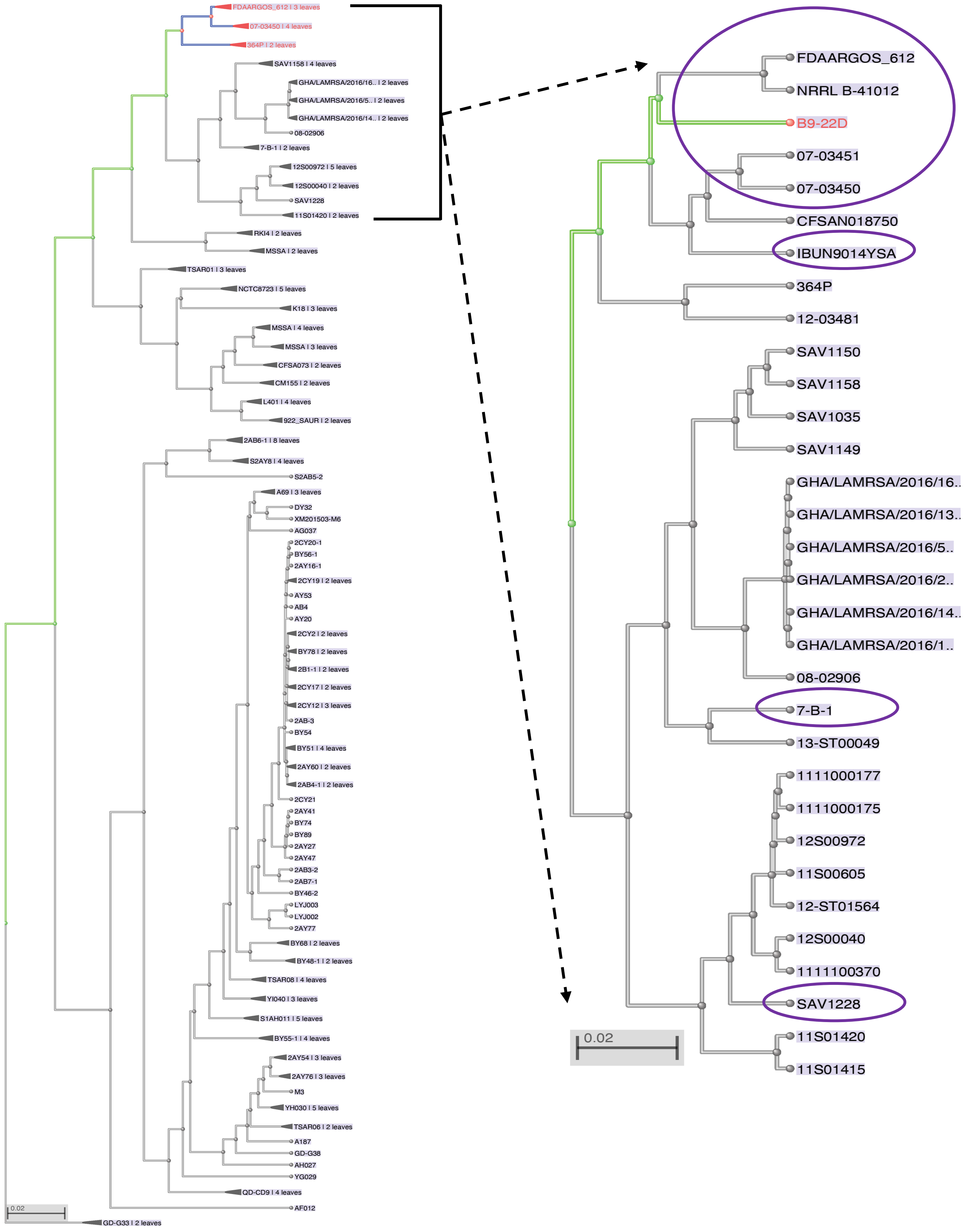

Fig. S4. Subtree of the phylogram (**Fig. S1**) that included retail pork isolate from our laboratory (strain name in red). Purple circles indicate the strains from retail pork source or pig source. Details of strains in the enlarged subset of subtree is listed in **Table S4**.

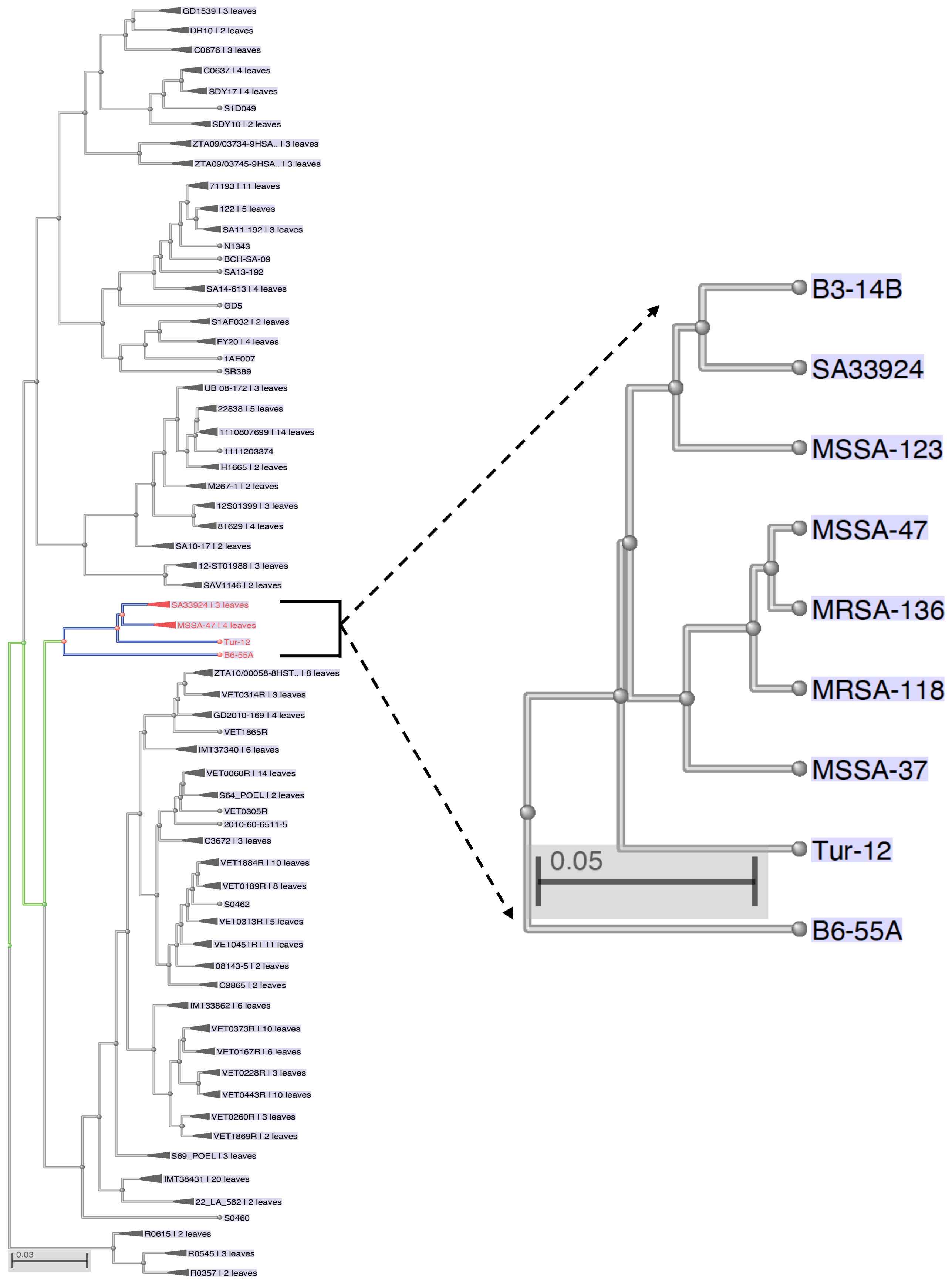

Fig. S5. Subtree of the phylogram (**Fig. S1**) that included retail turkey isolate from our laboratory (**B3-14B, B6-55A**). Details of strains in the enlarged subset of subtree is listed in **Table S5**.

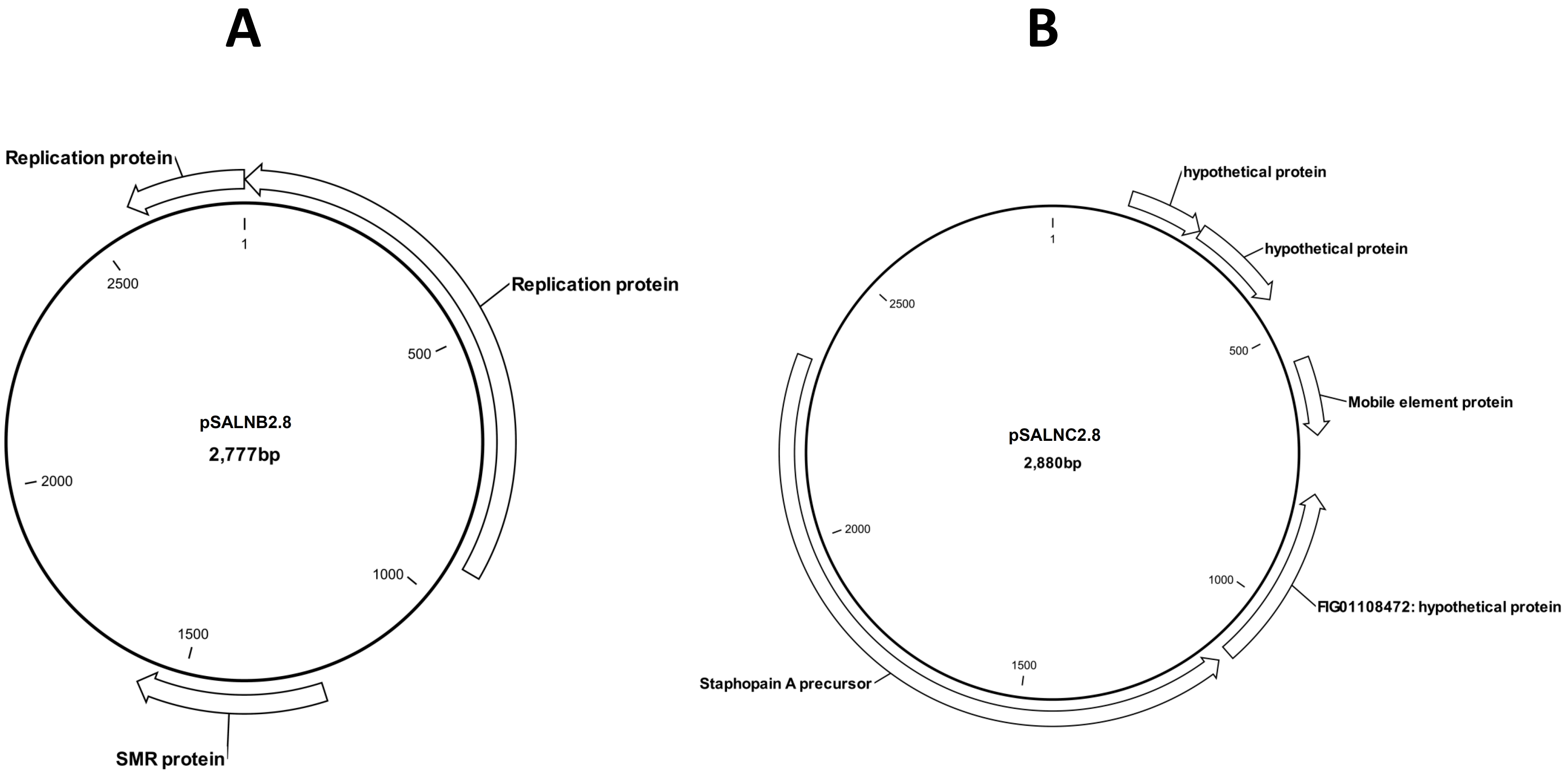

Figure S6. Maps of small plasmids A) pSALNB2.8 and B) pSALNC2.8.

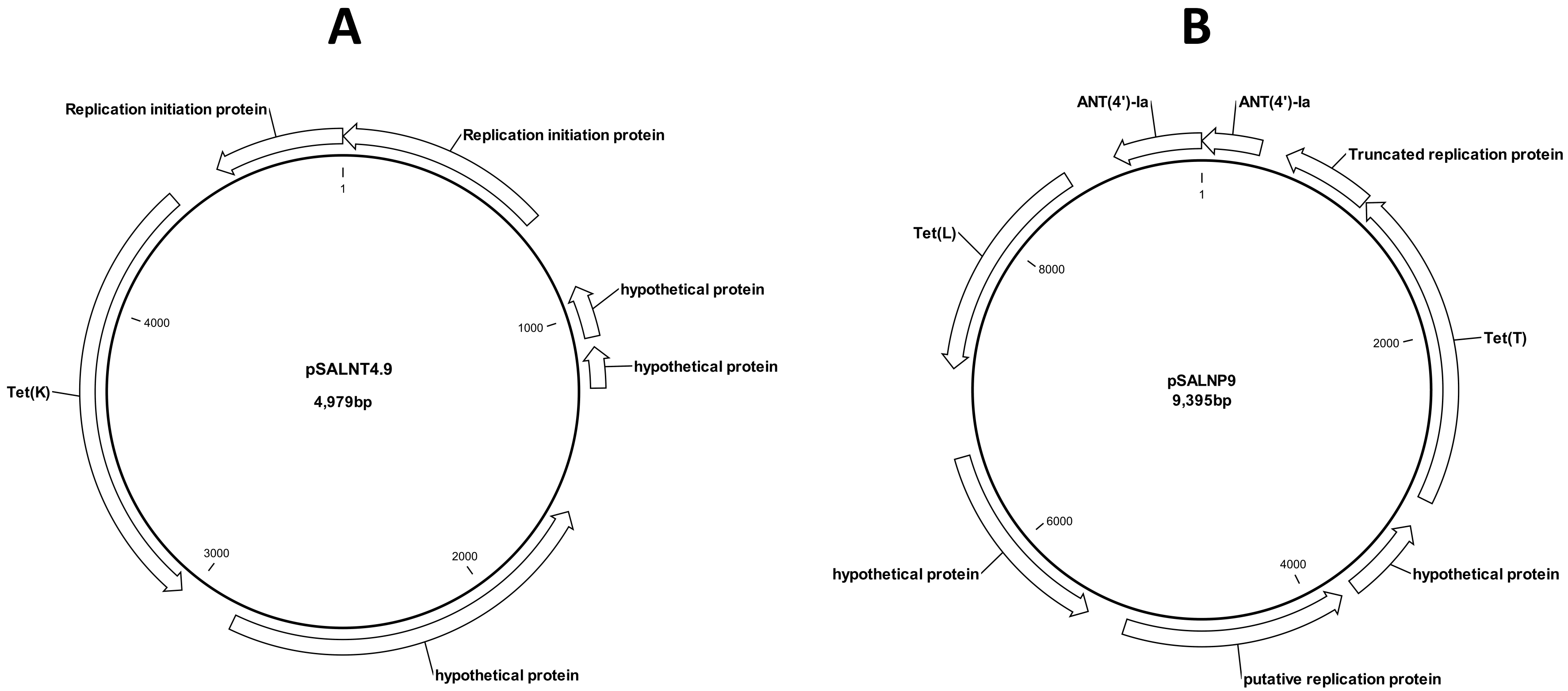

Figure S7. Map of plasmids A) pSALNT4.9 and B) pSALNP9 harboring tetracycline resistance genes.

A

B

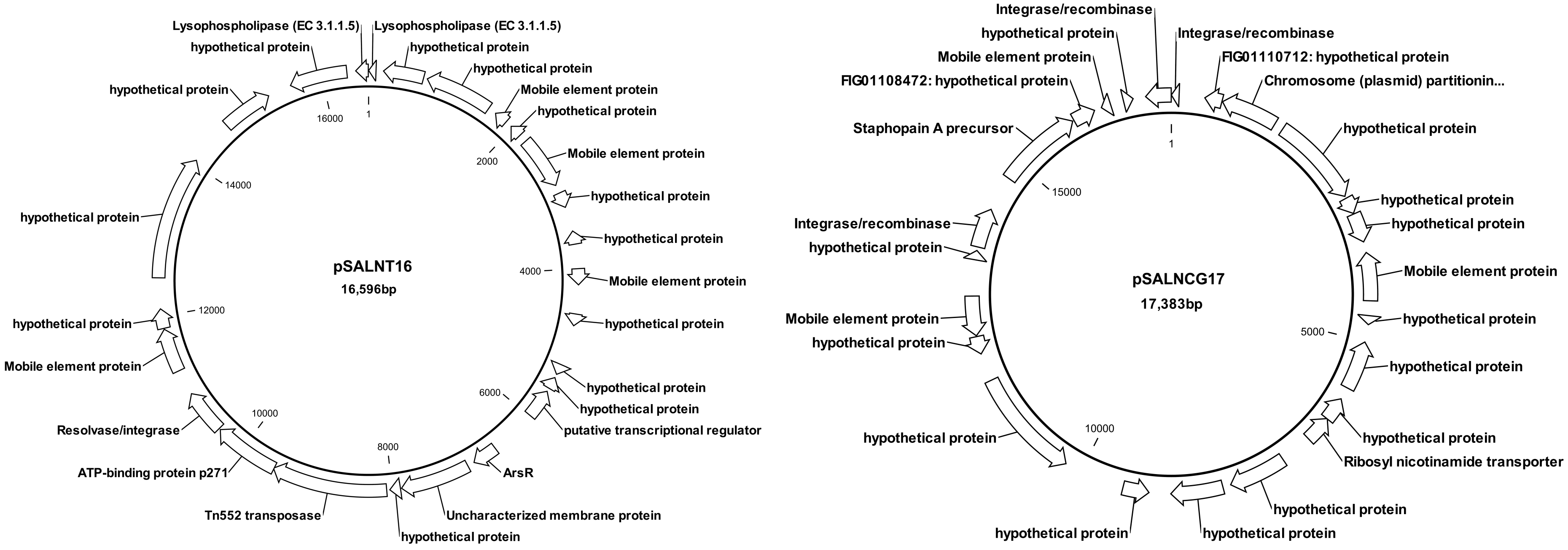

Figure S8. Map plasmids A) pSALNT16 and B) pSALNCG17. The sequence of other ~ 17 kb plasmids (pSALNCL17, pSALNC17, not shown) are like pSALNCG17.

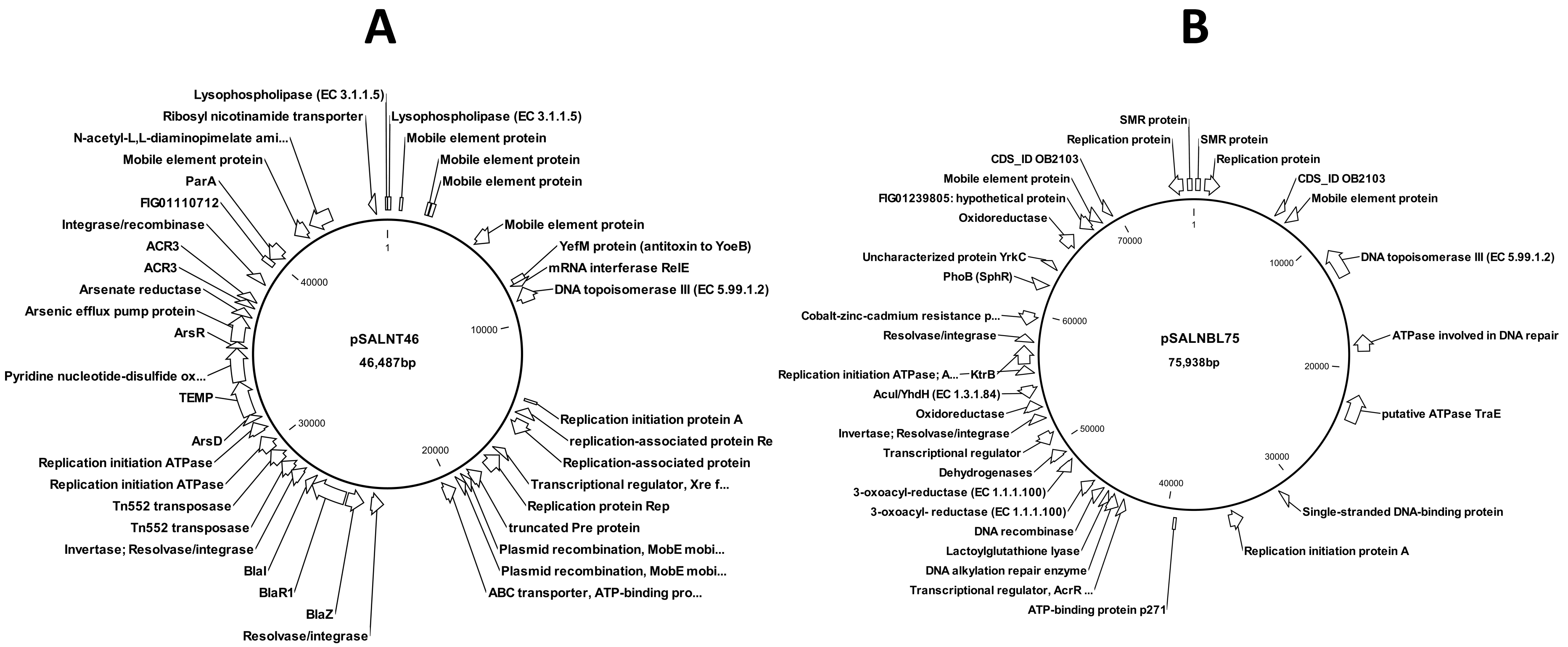

Figure S9. Map of two large plasmids(>45kb) A) pSaLNT46 and B) pSALNBL75. Hypothetical proteins are not shown.

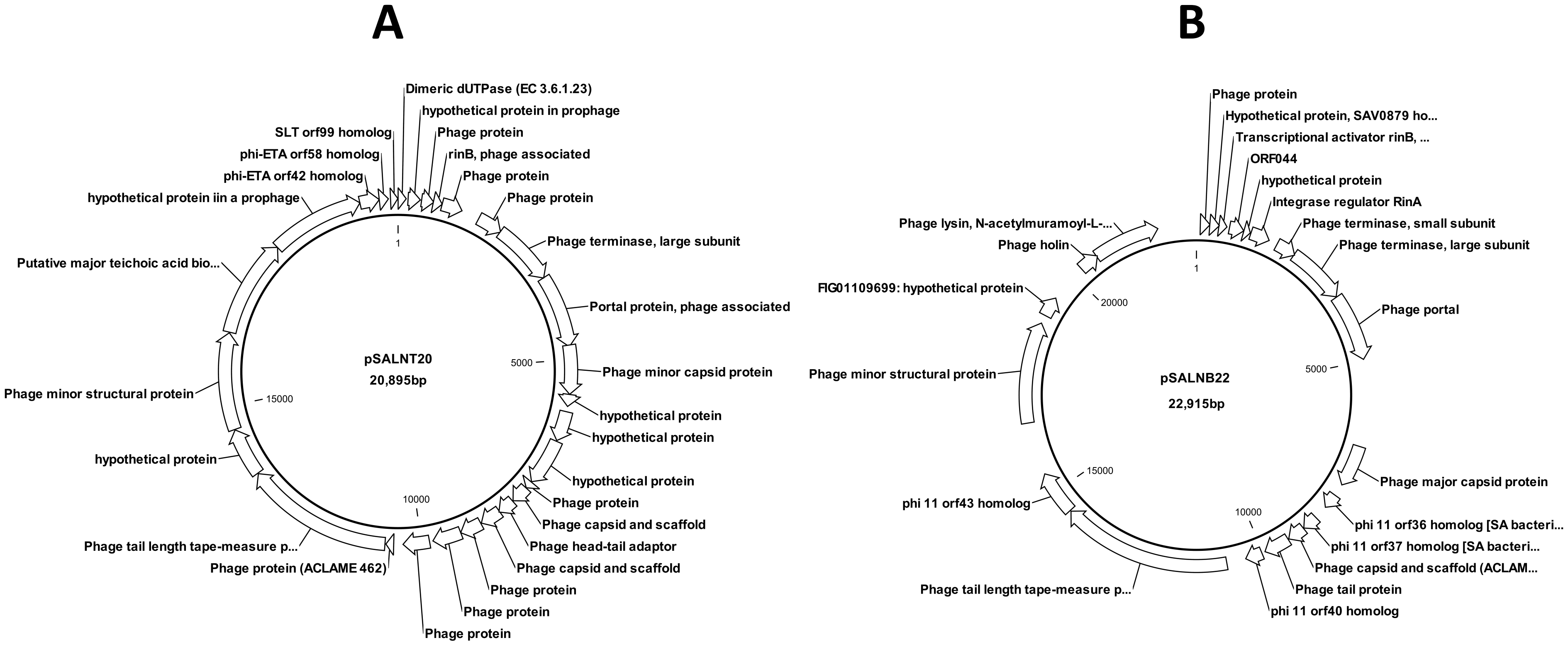

Figure S10. Map of plasmids A) pSALNT20 and B) pSALNB22, which harbor genes encoding phage like proteins.
